# The Impact of Cardiovascular Diseases on Postoperative Complications in Orthopedic Trauma Patients

**DOI:** 10.3390/diagnostics15131576

**Published:** 2025-06-20

**Authors:** Felix Erne, Larissa Mühlberger, Christoph Ihle, Sabrina Ehnert, Tina Histing, Andreas K. Nüssler, Elke Maurer

**Affiliations:** 1 Siegfried Weller Research Institute, BG Unfallklinik, Department of Trauma and Reconstructive Surgery, Eberhard Karls University Tuebingen, Schnarrenbergstr. 95, 72076 Tuebingen, Germany; 2 Department of Orthopedics and Traumatology, Diakonie-Klinikum Hospital, Rosenbergstr. 38, 70176 Stuttgart, Germany; 3 m&i-Fachkliniken Hohenurach, Orthopädische Klinik, Immanuel-Kant-Str. 33, 72574 Bad Urach, Germany; 4 Lumedis Orthopedics, Kirchstr. 14, 60311 Frankfurt, Germany

**Keywords:** cardiovascular diseases, postoperative complications, Clavien–Dindo classification, orthopedic trauma surgery, fractures of the upper and lower extremities

## Abstract

**Background:** Cardiovascular diseases (CVD) are recognized as a leading cause of morbidity and mortality in the population worldwide. A healthy cardiovascular system enables adaptation to trauma and physical stress. This study targets the reciprocal relation between CVD and postoperative complications after trauma surgery. **Methods:** In 2014, a randomized and standardized acquisition of study patients was initiated at a Level I trauma center in Germany. The burden of CVDs and the location, type, and severity of injuries were categorized. Clavien–Dindo classification was used to record postoperative complications. **Results:** The study analyzed a cohort of 1262 patients, representing a diverse range of orthopedic treatment disciplines within the hospital. It highlighted that patients with lower leg fractures experienced significantly higher complication rates, particularly linked to heart valve diseases and chronic venous insufficiency. Age and sex were not found to have a significant impact. Multivariate analysis identified additional key influencing factors for the development of complications, including the number of CVDs, nutritional status, smoking habits, and mobility levels. **Conclusions:** CVDs play a pivotal role in elevating complication rates post-trauma-surgery. Trauma-related extremity conditions are notably more severe when accompanied by CVD. A personalized approach that accounts for cardiovascular risk factors could significantly improve treatment outcomes in the future.

## 1. Introduction

The cardiovascular system (CVS) is a vital and important network for the homeostasis of the human body. It provides oxygen, nutrients, and metabolites to the tissues through the circulation of the blood. Cardiovascular risk factors (CVRF), such as obesity, high blood pressure, elevated cholesterol levels, smoking, and diabetes mellitus, are directly linked to more than half of all cardiovascular diseases (CVD) worldwide [1]. While age-standardized mortality rates have decreased in many regions, due to the rising absolute number of deaths globally, CVD remains a leading cause of all deaths worldwide [2]. A healthy CVS enables dynamic adaptation to external circumstances like trauma or stress. A compromised cardiovascular system can worsen health-related issues and cause adverse events. There is evidence that the high incidence of CVRF and CVD also influences the occurrence of postoperative complications in orthopedic surgery [3]. Injuries can be associated with direct vascular damage, indirect impaired blood flow, and long-term issues like wound healing disorders [4]. Orthopedic surgery places additional strain on the affected tissues [5]. Minimizing physical stress necessitates precise surgical techniques and comprehensive postoperative care. However, if postoperative complications arise, they can be classified according to the Clavien–Dindo classification (CDC). The system provides an objective, simple, and reproducible method for reporting negative events and has been extensively used and validated within clinical practice and research. The CDC categorizes postoperative complications based on the type of therapy required [6,7]. The advantage of this classification system is its focus on deviations from the planned treatment pathway rather than a detailed description of each operation. This approach allows the comparison of postoperative abnormalities across different types of surgery. Consequently, we performed a retrospective study on orthopedic trauma patients to assess the impact of CVD on complication rates using the CDC. Following the initial analysis of the entire orthopedic cohort, the specific subgroups will be analyzed as well, including patients with fractures in the upper and lower arms and legs. To ensure optimal care for patients with CVDs in trauma surgery, it is crucial to identify and evaluate their impact on potential adverse outcomes effectively [8,9,10]. This study aims to show the reciprocal relationship between CVD and trauma. Our study design is geared towards fully automated and AI-supported data collection in the future. All information sources can be used with little effort. In orthopedic trauma surgery, patients must be treated in their present state at the time of the accident. Addressing a diverse range of modifiable CVRF in cases of CVDs offers hope that medical interventions, beyond surgical procedures, may lead to improved outcomes.

## 2. Materials and Methods

### 2.1. Study Design

In 2014, a randomized and standardized acquisition of study patients was initiated at a Level I trauma center in Germany. The study cohort represents a broad cross-section of all treatment disciplines at the orthopedic trauma hospital. Patients consenting to participate in the study underwent a comprehensive assessment of risk factors for postoperative surgical complications. The screening encompassed both patients presented primarily after trauma and those who had received prior external treatment and were admitted in line with the treatment mandate. To avoid bias, patients who had undergone previous surgeries at external institutions were excluded. Detailed information can be found in the exclusion criteria.

### 2.2. Data Collection

The primary focus of this study is on the CVDs and their impact on the patients’ outcomes after trauma surgery. In our study, CVD status was determined through a combination of self-reporting and verification using available pre-treatment medical records. Confirmed diagnoses were documented where possible to enhance the validity of the data. Missing data were actively requested and retrieved, where possible, in order to improve the completeness and reliability of the dataset. CVD burden was categorized into the following conditions: hypertension, chronic heart disease (CHD), angina pectoris (AP), heart failure, heart valve disease, cardiac arrhythmia, infectious heart diseases, pulmonary heart diseases, cerebrovascular diseases and transient ischemic attack (TIA), peripheral artery disease (PAD), chronic venous insufficiency (CVI), and other CVD. Medications with a primary effect on the CVS, referred to as cardiovascular medication (CVM), were systematically recorded and categorized by their active ingredient groups.

In addition, this study recorded the following CVRFs: obesity, hypertension, smoking, and diabetes mellitus. Obesity was assessed by the body mass index (BMI), calculated by dividing the body weight in kilograms by the square of the height in meters. The WHO classification of obesity was applied [11]. Based on smoking status and history, the study cohort was categorized as non-smokers, active smokers, and former smokers. Diabetes mellitus was screened according to the guidelines of the American Diabetes Association [12].

Furthermore, the nutritional risk score (NRS) was calculated for each patient [13]. The NRS is a tool used to identify patients who may benefit from nutritional support, as they are at risk of malnutrition. Factors such as nutritional status, weight loss, BMI, food intake, and disease severity were used to calculate a score that guided clinical decision making [13,14].

The AO/OTA classification system was used to describe fractures’ location, type, and severity. The system provides a standardized method for precise diagnosis and treatment planning developed by the AO Foundation and the Orthopaedic Trauma Association [15]. The local tissue damage and the overall injury severity to the body were screened. All parameters were statistically analyzed to determine the homogeneity of the cohort.

### 2.3. Exclusion Criteria

Patients who received non-operative (conservative) treatment for orthopedic trauma were excluded from this analysis. Additionally, individuals who underwent elective surgical procedures for non-traumatic conditions, such as joint replacement surgeries, were not included. To further ensure consistency in treatment protocols, patients who had previously undergone surgery at external institutions and were referred to our center due to complications or the need for revision surgery were also excluded. These external procedures might not be performed according to our standardized clinical algorithms and could therefore not be reliably evaluated within the scope of this study. Excluding such cases helped to enhance the homogeneity of the study population and reduce potential sources of bias.

### 2.4. Classification of Postoperative Complication

To record postoperative complications, we applied the Clavien–Dindo classification (CDC), which provides a five-point-scale system based on the required therapy for treating complications. This system offers an objective, simple, and reproducible method for reporting negative surgical events and has been widely adopted and validated in clinical practice and research since its introduction in 2004 [6]. The grading system describes postoperative complications ranging from minor deviations (Grade I) to death (Grade V). Category I describes minor variations from the usual postoperative course not requiring any intervention, such as the use of antiemetics, antipyretics, analgesics, electrolytes, and physiotherapy. These are standard postoperative treatments and are not classified as complications in this analysis. Intermediate grades involve progressively greater levels of intervention, ranging from pharmacological treatments and blood transfusions (Grade II), to surgical or procedural interventions (Grade III), and life-threatening complications requiring intensive care (Grade IV) [7].

### 2.5. Statistics

All collected data were pseudonymized and imported into SPSS (IBM SPSS Statistics 25) for statistical analysis. The Shapiro–Wilk test was used to check for normal distribution. Data with normal distribution were analyzed using the *t*-test, while non-normally distributed data were analyzed using the two-sided Mann–Whitney U-test. Statistical significance was determined at an alpha level of 0.05. Multivariate regression analysis was conducted to assess the impact of CVDs and other related risk factors on the complication rate in orthopedic trauma patients. Data were adjusted for age, gender, and comorbid conditions to account for potential confounding factors. Furthermore, *p*-values were adjusted using the Bonferroni correction to address the issue of multiple comparisons. This adjustment has been applied consistently throughout the analysis. Significant results are displayed in bold.

## 3. Results

**Figure 1:**
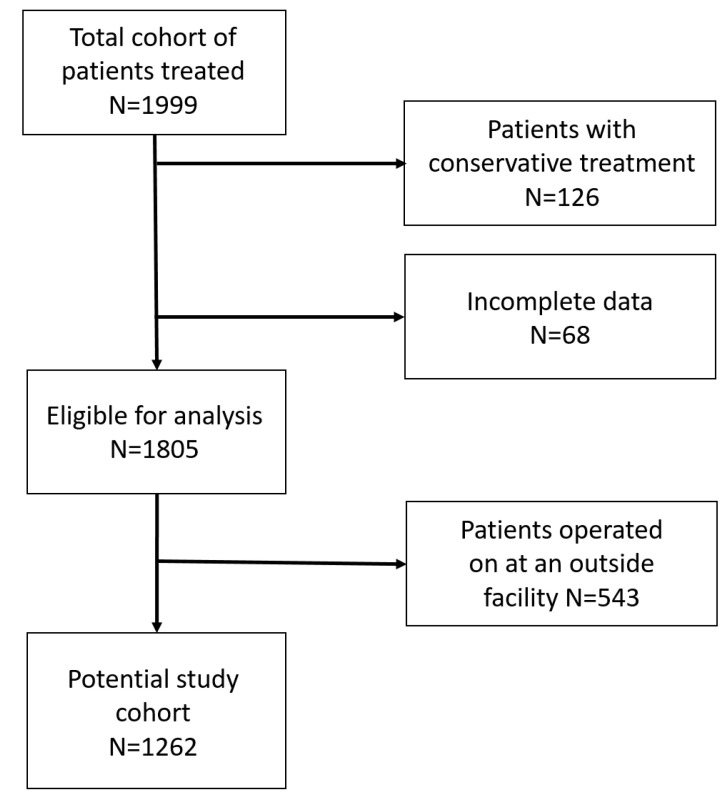
Flowchart for study cohort.

The structured database of the Siegfried Weller Research Institute comprised *N* = 1999 patient records. Of these, 194 patients were excluded from the statistical analysis, 68 of them due to incomplete data and 126 because conservative treatments were not considered in this study. A further 543 individuals from a total of 1805 patient records were excluded as they had been operated on at an outside facility. The result represents the final cohort of 1262 participants (Figure 1).

Based on the CDC, 643 patients were categorized as Category 0, whereas 291 patients were in Category I. Additionally, 135 patients fell into Category II, 122 into Category III, and 77 into Category IV, with only one patient classified as Category V. Patients in Categories 0 and I were grouped together since the use of antiemetics, antipyretics, analgesics, electrolytes, and physiotherapy—standard postoperative treatments for most patients— were not considered as complications in our cohort.

The demographic data were derived for the total cohort and for the subgroups defined according to the fracture location. Demographic data for patients with upper and lower arm fractures, as well as those with femur and tibia fractures, are presented in Table 1.

The univariate analysis of the total cohort reveals a significant impact of CVDs on the complication rate. Specifically, chronic heart disease (CHD), heart failure, heart valve disease, cardiac arrhythmia, pulmonary heart disease, peripheral artery disease (PAD), and CVI all significantly influence the complication rate (Table 2).

Pre-existing CVD did not show an association with complication rates in patients with upper arm fractures (Table 3). Similarly, patients with pre-existing CVD did not show an association with complications in lower arm fractures, except for cardiac arrhythmia, which was associated with elevated complication rates (Table 4).

Patients with upper arm fractures did not show a significantly higher complication rate in the presence of pre-existing CVD compared to those without such conditions (Table 3). Similarly, patients with lower arm fractures showed no significant increase in complications associated with CVDs, except for cardiac arrhythmia, which was found to significantly elevate the complication rate (Table 4).

CVDs did not appear to significantly correlate with the complication rate in patients with femur fractures (Table 5). In contrast, patients with lower leg fractures showed a notable association between increased complication rates and the presence of heart valve diseases and CVI (Table 6).

In a multivariate analysis, we tested for additional factors that might influence the development of complications. Complication rate was not associated with age or sex in our cohort. However, in the overall cohort, a higher number of CVDs, smoking habits, poor nutritional status, and reduced mobility were associated with a higher likelihood of complications. Considering the subgroups (upper arm, lower arm, femur, lower leg), it can be observed that the number of CVDs significantly affected patients with lower arm and lower leg fractures. The impact of smoking on the complication rate was significantly higher in patients with lower leg fractures but was slightly lower in those with lower arm and femur fractures. Less mobility significantly negatively impacted the complication rate in patients with lower leg fractures but was less significant in those with femur fractures. Nutritional status appeared to be more relevant for patients with fractures of the lower extremities, though it did not reach statistical significance (Table 7 and Table 8).

## 4. Discussion

In our cohort, univariate analysis revealed significant associations between CVD and overall complication rates. While upper arm fractures generally did not show a significant correlation with complications in the presence of CVD, cardiac arrhythmias were associated with a higher complication rate in forearm fractures. In cases of lower leg fractures, valvular heart disease and CVI were likewise associated with increased complication rates. It is important to note that these findings reflect statistical associations rather than causal relationships.

The CVS plays a vital role in wound healing by delivering oxygen and nutrients essential for tissue repair. However, CVD can impair blood flow, resulting in prolonged inflammation, increased susceptibility to infection, and delayed recovery [16]. A strong correlation exists between peripheral fractures in the extremities, CVD, and complication rates [16]. The ‘last meadow’ theory, which postulates that areas with terminal arteries are the most susceptible to inadequate nutrient supply, would support these findings. Considering the hydrostatic pressure and body weight as the additional load on the legs, it is expected that the existent CVD raises the complication rate. The increased risk of complications in conditions that impair venous return, such as CVI, supports this observation. Effective blood flow from the extremities depends not only on a healthy cardiovascular system but also on sufficient mobility, as physical activity activates the muscular venous pump. The statistical significance of reduced mobility as a key factor in the complication rate may explain this association [17,18,19]. While anticoagulant medication is vital for preventing thromboembolic events, it also significantly increases the risk of bleeding complications [3,17]. This delicate balance is reflected in the higher vulnerability of patients with anticoagulant therapy undergoing orthopedic procedures [17]. The literature research shows that patients with pulmonary hypertension (PH) experience increased perioperative morbidity and mortality when undergoing anesthesia and major surgeries [6]. The risks associated with PH become higher under such conditions as stress, pain, mechanical ventilation, and trauma-induced inflammation [18]. Patients with complex CVI face a higher risk of complications following total knee arthroplasty than those with milder forms of the condition [19].

Multivariate analysis also demonstrated that, in addition to CVDs, factors such as smoking habits, nutritional status, and mobility were significantly associated with complication rates, particularly in patients with lower limb fractures. These results reflect statistical associations and should not be interpreted as evidence of causation. Tobacco consumption has been linked to increased inpatient trauma morbidity, additional oxidative stress being supposedly one of the underlying reasons [20]. Screening reveals a significantly higher complication rate in smoking patients with peripheral extremity fractures [20,21]. Smokers face an elevated risk of deep wound infections, wound dehiscence, and related re-operations and re-admissions following ankle fracture surgery [21]. Our findings are consistent with the existing literature, which suggests that cigarette smoke has a greater impact on the peripheral extremities [20,22,23]. This effect is most probably be attributed to the significant impact of cigarette-induced vasoconstriction on the small blood vessels, boosting complications in distal areas [22,23]. Our data also demonstrates that limited mobility has particularly detrimental impact in the lower extremities. In this context, Freigang et al. note with concern that reduced mobility is associated with restrictions in one’s independence, cognitive decline, falls, and increased mortality [24]. Additionally, patients with deficient mobility or prolonged bed rest, associated with skin sores, infections, delayed healing, and a higher incidence of venous thromboembolism, are more likely to develop fracture complications [25]. Malnutrition can be regarded as another cause linked to negative outcomes in hospitalized patients, including higher complication rates, prolonged stays, and mortality [26]. Among geriatric trauma patients, 7–62% were malnourished (Maurer, 2020 [25]). Malnutrition in orthopedic trauma patients with surgical site infections is associated with more comorbidities and complications [27]. Nutritional intervention is cost effective and enhances recovery [28]. Given its impact, addressing malnutrition and CVD is crucial for improving patient outcomes, particularly with the lower extremities’ fractures. Neither age nor sex had a significant correlation with complication rates in our cohort. In this respect, our study data are consistent with the results of other publications [24,25,26,27].

In our study design, we used the existing medical records, which may have gaps or inconsistencies, potentially leading to incomplete data and affecting our analysis accuracy. Simple randomization was applied, balanced by the long study period and high patient number. However, small subgroup sizes for specific fracture regions, except for the group with lower leg fractures (279 patients), set limits on the data accuracy and reliability. Larger samples from other fracture locations would improve our findings.

Healthcare systems worldwide are adopting person-centered care to enhance performance [28]. In 2021, a systematic review investigated the effects of patient engagement interventions for older patients with multimorbidity and found improvements in health and patient-reported outcomes, such as higher quality-adjusted life-years, fewer hospital visits, and disease-specific symptoms [29]. The establishment of patient-centered treatment approaches is also urgently needed considering the consequences of postoperative complications. For example, patients with proximal femoral fractures are a particularly well-studied group. In a cross-sectional observational study with 63,778 patients, 12.6% died in the 90 days after admission, and a further 10.0% died by day 365 [30]. The future approach in our clinic is to establish an early warning system for possible complications. In the future, this system should calculate the individual risk of postoperative complications based on the available treatment records. All necessary information can be generated automatically from the treatment and billing documents. By using this approach, it would be possible, for example, to allocate a higher staffing ratio to vulnerable patient groups, to be able to argue better against the burden of higher costs, and to create a basis for further interventions. There are numerous and promising intervention options for achieving a positive effect on the patient population under investigation. Cardiac arrhythmias are one of the most common cardiac diseases and are very accessible to interventions. In primary care, the optimal approach to the diagnosis of atrial fibrillation is not quite achievable [31]. However, anesthesiologic care during trauma surgery offers the unique opportunity to monitor electrical activity in the heart. The additional automated calculation of the CHA2DS2-VASc score could also increase the detection rate of previously silent cardiac arrhythmias [31]. A cardiological treatment should be the rule. CVI is a progressive vascular condition characterized by venous hypertension and chronic inflammation, leading to significant clinical and socioeconomic impacts [32]. If the high combined risk is known, it makes sense to treat this disease directly. In addition to just-in-time diagnostic clarification, there is also the possibility of drug intervention and the integration of this disease into physiotherapeutic follow-up treatment concepts [32,33,34]. The global burden of valvular heart disease is expected to steadily increase, and there is an association with major cardiovascular events such as heart failure, arrhythmia, and early mortality [35]. Therefore, increased attention is also required in non-cardiology departments. A structured screening could be a strategy to mitigate the progression of VHD and to decrease costs in healthcare systems [35]. In particular, early detection, personalized management, and cutting-edge interventions are described to optimize outcomes [36]. Directly addressing these diseases could have a positive effect, both in trauma surgery and in cardiology.

To support clinical decision making, we propose a predictive framework integrating the key risk factors identified in our study—number of cardiovascular conditions, smoking status, mobility level, and nutritional status. These variables could form the basis of a scoring system to estimate a patient’s individual risk of postoperative complications. Such a tool may assist in early identification of high-risk patients and enable targeted perioperative management. Future research should validate this approach prospectively and refine the weighting of each factor to optimize accuracy and clinical utility. This concept aligns with person-centered care and promotes preventive strategies in orthopedic trauma surgery.

## 5. Conclusions

CVDs appear to be significantly associated with complication rates following trauma surgery. Our findings suggest that trauma-related conditions in the extremities may be more complex when coexisting with CVD. In our cohort, the upper extremities showed a higher association with cardiac arrhythmias, while the lower extremities were more frequently affected by conditions impairing venous return. We recommend that CVDs be carefully considered during diagnostics and treatment planning in orthopedic trauma surgery, as these patients may require special attention. From a public health perspective, screening for CVRFs remains important and should be promoted. Patients identified with CVRFs should receive appropriate follow-up and support in optimizing modifiable risk factors.

## Figures and Tables

**Table 1:** Demographic and descriptive data for the total cohort and subgroups of patients with upper and lower arm, femur, and tibia fractures.

Characteristics	All	Upper Arm	Lower Arm	Femur	Tibia
	N = 1262	N = 94	N = 155	N = 119	N = 297
Age (years)	57.2 ± 17.6	59.3 ± 16.3	53.1 ± 18.0	65.3 ± 19.2	50.1 ± 16.0
Men	655 (51.9%)	37 (39.4%)	62 (40.0%)	305 (65.3%)	305 (65.3%)
Size (cm)	171.5 ± 9.6	169.2 ± 9.2	171.6 ± 9.0	171.1 ± 10.9	173.7 ± 8.9
BMI (kg/m^2^)	27.1 ± 5.0	26.5 ± 4.9	26.6 ± 4.9	25.3 ± 4.8	27.2 ± 4.8
Smoking status					
Non-smoker	681 (54.0%)	55 (58.5%)	99 (63.9%)	68 (57.1%)	144 (48.5%)
Smoker	581 (46.0%)	39 (41.5%)	56 (36.1%)	51 (42.9%)	153 (51.5%)
Mobility					
Unrestricted	1028 (81.5%)	83 (88.3%)	151 (97.4%)	75 (63.0%)	251 (84.5%)
Restricted	234 (18.5%)	11 (11.7%)	4 (2.6%)	44 (37.0%)	46 (15.5%)
CCI					
0 points	885 (70.1%)	71 (75.5%)	135 (87.1%)	57 (47.9%)	236 (79.5%)
1–2 points	267 (21.2%)	19 (20.2%)	17 (11.0%)	43 (36.1%)	45 (15.2%)
3–4 points	82 (6.5%)	3 (3.2%)	2 (1.3%)	13 (10.9%)	9 (3.0%)
>4 points	28 (2.2%)	1 (1.1%)	1 (0.6%)	6 (5.0%)	7 (2.4%)
NRS					
NRS < 3	1056 (83.7%)	78 (83.5%)	144 (92.9%)	76 (63.9%)	256 (86.2%)
NRS ≥ 3	205 (16.3%)	15 (16.5%)	11 (7.1%)	43 (36.1%)	41 (13.8%)
Average number of comorbidities	2.5 ± 2.5	2.2 ± 1.9	1.5 ± 1.8	1.4 ± 1.7	1.7 ± 2.0
Average number of cardiovascular comorbidities	0.9 ± 1.2	0.7 ± 1.0	0.5 ± 9.6	3.1 ± 2.7	0.6 ± 1.0
Medication intake	790 (62.6%)	63 (67.0%)	71 (45.8%)	87 (73.1%)	143 (48.1%)
Complication classification	328 (26.0%)	21 (22.3%)	24 (15.5%)	51 (42.9%)	74 (24.9%)
Clavien–Dindo					
Clavien–Dindo 0	643 (51.0%)	39 (41.5%)	85 (54.8%)	49 (41.2%)	153 (51.5%)
Clavien–Dindo I	291 (23.0%)	34 (36.2%)	48 (29.7%)	19 (16.0%)	70 (23.6%)
Clavien–Dindo II	135 (10.7%)	10 (10.6%)	9 (5.8%)	25 (21.0%)	32 (10.8%)
Clavien–Dindo III	122 (9.7%)	9 (9.6%)	12 (7.7%)	8 (6.7%)	35 (11.8%)
Clavien–Dindo IV	70 (5.5%)	1 (2.1%)	3 (1.9%)	18 (15.1%)	7 (2.4%)
Clavien–Dindo V	1 (0.1%)	0 (0.0%)	0 (0.0%)	0 (0.0%)	0 (0.0%)

Data are given as mean (±SD) or number (percentage).

**Table 2:** Univariate analysis of the impact of CVDs on the complication rate of the total cohort of orthopedic trauma patients.

Cardiovascular Disease	All	No Complication	Complication	*p*-Value
	N = 1262	N = 934	N = 328	
Hypertension	507 (40.2%)	365 (39.1%)	142 (43.3%)	0.19
CHD/AP	111 (8.9%)	66 (7.1%)	45 (13.7%)	**<0.001**
Heart failure	76 (6.0%)	42 (4.5%)	34 (10.4%)	**<0.001**
Heart valve disease	52 (4.1%)	29 (3.1%)	23 (7.1%)	**0.003**
Cardiac arrhythmia	113 (9.0%)	67 (7.2%)	46 (14.0%)	**<0.001**
Infectious heart diseases	4 (0.3%)	1 (0.1%)	3 (1.0%)	0.06
Pulmonary heart diseases	28 (2.2%)	15 (1.6%)	13 (4.0%)	**0.017**
Cerebrovasc. diseases/TIA	48 (3.8%)	30 (3.2%)	18 (5.5%)	0.09
PAD	40 (3.2%)	24 (2.6%)	16 (4.9%)	**0.045**
CVI	76 (6.0%)	41 (4.4%)	35 (10.7%)	**<0.001**
Other cardiovasc. diseases	7 (0.6%)	4 (0.4%)	3 (1.0%)	0.08

Data are given as a number (percentage).

**Table 3:** Univariate analysis of the impact of CVDs on the complication rate of orthopedic trauma patients with a fracture of the upper arm.

Cardiovascular Disease	Upper Arm	No Complication	Complication	*p*-Value
	N = 94	N = 73	N = 21	
Hypertension	37 (39.2%)	28 (38.4%)	9 (42.9%)	0.80
CHD/AP	9 (9.6%)	7 (9.6%)	2 (9.5%)	1.00
Heart failure	7 (7.4%)	5 (6.8%)	2 (9.5%)	0.65
Heart valve disease	1 (1.0%)	1 (1.4%)	0 (0.0%)	1.00
Cardiac arrhythmia	9 (9.6%)	6 (8.2%)	3 (14.3%)	0.41
Infectious heart diseases	0 (0.0%)	0 (0.0%)	0 (0.0%)	-
Pulmonary heart diseases	1 (1.0%)	0 (0.0%)	1 (4.8%)	0.22
Cerebrovasc. diseases/TIA	0 (0.0%)	0 (0.0%)	0 (0.0%)	-
pAVD	1 (1.0%)	1 (1.4%)	0 (0.0%)	1.00
CVI	3 (3.2%)	3 (4.1%)	0 (0.0%)	1.00
Other cardiovasc. diseases	0 (0.0%)	0 (0.0%)	0 (0.0%)	-

Data are given as a number (percentage).

**Table 4:** Univariate analysis of the impact of CVDs on the complication rate of orthopedic trauma patients with a fracture of the lower arm.

Cardiovascular Disease	Lower Arm	No Complication	Complication	*p*-Value
	N = 155	N = 131	N = 24	
Hypertension	43 (27.7%)	35 (26.7%)	8 (33.3%)	0.62
CHD/AP	5 (3.2%)	5 (3.8%)	0 (0.0%)	1.00
Heart failure	2 (1.3%)	2 (1.5%)	0 (0.0%)	1.00
Heart valve disease	2 (1.3%)	1 (0.8%)	1 (4.2%)	0.29
Cardiac arrhythmia	6 (3.9%)	3 (2.3%)	3 (12.5%)	**0.048**
Infectious heart diseases	1 (0.6%)	1 (0.8%)	0 (0.0%)	1.00
Pulmonary heart diseases	3 (1.9%)	1 (0.8%)	2 (8.3%)	0.40
Cerebrovasc. diseases/TIA	2 (1.3%)	1 (0.8%)	1 (4.2%)	0.29
pAVD	1 (0.6%)	1 (0.8%)	0 (0.0%)	1.00
CVI	5 (3.2%)	4 (3.1%)	1 (4.2%)	0.57
Other cardiovasc. diseases	0 (0.0%)	0 (0.0%)	0 (0.0%)	-

Data are given as a number (percentage).

**Table 5:** Univariate analysis of the impact of CVDs on the complication rate of orthopedic trauma patients with a fracture of the femur.

Cardiovascular Disease	Femur	No Complication	Complication	*p*-Value
	N = 119	N = 68	N = 51	
Hypertension	59 (49.6%)	34 (50.0%)	25 (49.0%)	1.00
CHD/AP	24 (20.2%)	10 (14.7%)	14 (27.5%)	0.11
Heart failure	16 (13.4%)	8 (12.5%)	8 (15.7%)	0.59
Heart valve disease	10 (8.4%)	4 (5.9%)	6 (11.8%)	0.32
Cardiac arrhythmia	21 (17.6%)	9 (13.2%)	12 (23.5%)	0.16
Infectious heart diseases	2 (1.7%)	0 (0.0%)	2 (3.9%)	0.18
Pulmonary heart diseases	3 (2.5%)	1 (1.5%)	2 (3.9%)	0.58
Cerebrovasc. diseases/TIA	8 (6.7%)	4 (5.9%)	4 (7.8%)	0.72
pAVD	6 (5.0%)	3 (4.4%)	3 (5.9%)	1.00
CVI	9 (7.6%)	4 (5.9%)	5 (9.8%)	0.50
Other cardiovasc. diseases	1 (0.1%)	0 (0.0%)	1 (2.0%)	0.42

Data are given as a number (percentage).

**Table 6:** Univariate analysis of the impact of CVDs on the complication rate of orthopedic trauma patients with a fracture of the lower leg.

Cardiovascular Disease	Lower Leg	No Complication	Complication	*p*-Value
	N = 279	N = 223	N = 74	
Hypertension	79 (28.3%)	56 (25.2%)	23 (31.1%)	0.36
CHD/AP	15 (5.4%)	8 (3.6%)	7 (9.5%)	0.06
Heart failure	10 (3.6%)	5 (2.2%)	5 (6.8%)	0.13
Heart valve disease	9 (3.2%)	4 (1.8%)	5 (6.6%)	**0.046**
Cardiac arrhythmia	15 (5.4%)	9 (4.0%)	6 (8.1%)	0.22
Infectious heart diseases	0 (0.0%)	0 (0.0%)	0 (0.0%)	-
Pulmonary heart diseases	3 (1.1%)	1 (0,4%)	2 (2.7%)	0.15
Cerebrovasc. diseases/TIA	6 (2.2%)	4 (1.8%)	2 (2.7%)	0.64
pAVD	9 (3.2%)	5 (2.2%)	4 (5.4%)	0.23
CVI	15 (5.4%)	7 (3.1%)	8 (10.8%)	**0.015**
Other cardiovasc. diseases	1 (0.4%)	1 0.4%)	0 (0.0%)	1.00

Data are given as a number (percentage).

**Table 7:** Multivariate analysis of the impact of CVDs on the complication rate of orthopedic trauma patients including other CVRF (upper extremities).

Characteristics	All N = 1262		Upper Arm N = 94		Lower Arm N = 155	
	Odds Ratio (95% KI)	*p*-Value	Odds Ratio (95% KI)	*p*-Value	Odds Ratio (95% KI)	*p*-Value
Age (years)	1.00 (0.99–1.01)	0.72	1.00 (0.96–1.03)	0.85	0.99 (0.96–1.02)	0.43
Sex (male)	1.06 (0.80–1.40)	0.68	1.23 (0.40–3.16)	0.82	0.71 (0.25–2.02)	0.52
No of cardiovascular diseases	1.21 (1.08–1.37)	**0.002**	1.19 (0.64–2.22)	0.58	2.03 (1.12–3.67)	**0.019**
Smoker	1.56 (1.20–2.04)	**<0.001**	1.02 (0.38–2.78)	0.97	2.40 (0.93–6.25)	0.07
NRS	1.75 (1.24–2.48)	**0.002**	1.32 (0.30–5.77)	0.71	0.520 (0.49–5.47)	0.59
Mobility	1.65 (1.19–2.30)	**0.003**	0.53 (0.08–3.48)	0.51	1.00 (0.00–0.00)	1.00

**Table 8:** Multivariate analysis of the impact of CVDs on the complication rate of orthopedic trauma patients including other CVRF (lower extremities).

Characteristics	All N = 1262		Femur N = 119		Lower Leg N = 279	
	Odds Ratio (95% KI)	*p*-Value	Odds Ratio (95% KI)	*p*-Value	Odds Ratio (95% KI)	*p*-Value
Age (years)	1.00 (0.99–1.01)	0.72	1.01 (0.98–1.04)	0.46	0.99 (0.97–1.01)	0.32
Sex (men)	1.06 (0.80–1.40)	0.68	1.54 (0.59–3.98)	0.38	1.05 (0.58–1.90)	0.88
No. of cardiovascular diseases	1.21 (1.08–1.37)	**0.002**	1.05 (0.78–1.40)	0.76	1.44 (1.07–1.94)	**0.015**
Smoker	1.56 (1.20–2.04)	**<0.001**	2.01 (0.82–4.96)	0.13	2.06 (1.16–3.64)	**0.013**
NRS	1.75 (1.24–2.48)	**0.002**	1.49 (0.61–3.64)	0.36	1.89 (0.88–4.04)	0.10
Mobility	1.65 (1.19–2.30)	**0.003**	2.18 (0.90–5.24)	0.08	2.08 (1.01–4.26)	**0.046**

## Data Availability

The datasets used and/or analyzed during the current study are available from the corresponding author on reasonable request.
